# Alignment adjustment using the Valgus stress technique can increase the surgical accuracy of novice surgeons during medial opening-wedge high Tibial osteotomy

**DOI:** 10.1186/s12891-021-04475-3

**Published:** 2021-06-25

**Authors:** Man Soo Kim, In Jun Koh, Yong Gyu Sung, Dong Chul Park, Sung Bin Han, Yong In

**Affiliations:** 1 Department of Orthopaedic Surgery, Seoul St. Mary’s Hospital, College of Medicine, The Catholic University of Korea, 222, Banpo-daero, Seocho-gu, Seoul, 06591 Republic of Korea; 2 Department of Orthopaedic Surgery, Eunpyeong St. Mary’s Hospital, College of Medicine, The Catholic University of Korea, 1021, Tongil Ro, Eunpyeong-gu, Seoul, 03312 Republic of Korea

**Keywords:** Novice, Expert, High Tibial osteotomy, Alignment, Correction, Accuracy, Weight-bearing line

## Abstract

**Background:**

The purpose of this study was to compare the degree of accuracy of coronal alignment correction with use of the “alignment adjustment under valgus stress technique” between expert and novice surgeons during medial opening-wedge high tibial osteotomy (MOWHTO).

**Methods:**

Forty-eight patients who underwent MOWHTO performed by an expert surgeon (expert group) and 29 by a novice surgeon (novice group) were enrolled in analysis. During surgery, lower-extremity alignment was corrected using the “alignment adjustment under valgus stress technique”. Normocorrection was defined as a weight-bearing line ratio between 55 and 70% and the correction accuracy was compared between expert and novice groups using the ratio of normocorrection to outliers. The clinical outcomes were also compared using the Western Ontario and McMaster Universities Osteoarthritis Index (WOMAC) at 1 year after surgery.

**Results:**

The undercorrection rate was 14.6% in the expert group and 13.8% in the novice group, while the overcorrection rate was 2.1% in the expert group and 3.4% in the novice group. In the ratio of normocorrection to outliers, no difference was found between the two groups at the one-year follow-up visit (83.3% in the expert group vs. 82.8% in the novice group; *p* > 0.05). Also, no significant differences were seen in WOMAC subscores immediately preoperatively and at 1 year after surgery (all *p* > 0.05).

**Conclusion:**

Adhering to the “alignment adjustment under valgus stress technique” protocol enabled novice surgeons to achieve similar surgical accuracy as that of an expert surgeon in coronal alignment during MOWHTO.

**Level of evidence:**

Level III.

## PACS Picture Archiving and Communication System

WBL Weight-bearing line

MA Mechanical axis

ICC Intraclass correlation coefficient

MPTA Medial proximal tibial angle

LDFA Lateral distal femoral angle

JLCA Joint line convergence angle

## Background

Medial opening-wedge high-tibial osteotomy (MOWHTO) is a widely used surgical treatment for medial compartment osteoarthritis (OA) of the knee with a varus deformity [1, 2], as well as isolated chondral defects in the medial compartment of the varus knee [3], osteonecrosis in the medial compartment [4], and posttraumatic arthritis and deformity [5, 6]. It shifts the weight-bearing load from the affected medial compartment to the relatively intact lateral compartment [7]. Proper correction of the deformity is essential to attain satisfactory clinical results after MOWHTO [8]. Under- or overcorrection can lead to inferior clinical outcomes [9, 10].

Surgical competence is paramount in MOWHTO to achieve satisfactory outcomes for patients [11]. Although every novice surgeon desires to refine their surgical skills in a short period of time and reduce the learning curve as much as possible in the field of surgery, the accumulation of surgical experience by way of practice is inevitably necessary to reach an adequate level of surgical competence [12]. In MOWHTO, it was reported that a learning curve of approximately 27 cases is necessary to avoid undercorrection [13]. Conversely, in the case of overcorrection, even if 100 cases were completed, adequate prevention was not possible [13]. As such, it can be difficult to attain appropriate alignment even with sufficient surgical experience in MOWHTO [13].

Various preoperative and intraoperative methods have been used to obtain the proper correction for MOWHTO, including the use of a mathematical formula [14], whole lower-extremity radiographs [15], electrocautery cables [16, 17], grids [18], fluoroscopy [16, 17], imaging software [19], patient-specific instruments [20], and a navigation system [21]. Despite these efforts, however, some number of outlier cases occur continuously after MOWHTO because alignment corrections made in the supine position cannot exactly represent the postoperative weight-bearing alignment. Recently, the “alignment adjustment under valgus stress technique” protocol to reduce outliers during MOWHTO has been introduced [22]. In this context, after the angle is corrected using the Dugdale method [23], limb alignment is finally adjusted using the intraoperative cable technique by applying valgus stress to the knee joint. Although the reporting authors noted a high accuracy rate [22], questions persist as to whether the manual valgus stress during MWOHTO could show consistency and reproducibility, in particular, for cases treated by a novice surgeon.

In the light of recently gained knowledge, the purpose of this study was to compare the degree of accuracy of surgical correction with use of the “alignment adjustment under valgus stress technique” during MOWHTO between expert and novice surgeons. It was hypothesized that the “alignment adjustment under valgus stress technique” protocol would result in similar acceptable accuracy levels of surgical performance among both expert and novice surgeons during MOWHTO.

## Methods

From June 2018 to November 2019, a total of 84 MOWHTO procedures were performed by either an expert surgeon (*n* = 52 cases, expert group) and a novice surgeon (*n* = 32 cases, novice group) using a contemporary locking plate at a single institution. The expert surgeon had worked as a knee surgeon for 20 years and performed more than 40 MOWHTOs annually for more than 6 years. In contrast, the novice surgeon had completed a basic four-year orthopedic residency and three-year knee and sports medicine fellowship but lacked experience as an operator of MOWHTO. This study was approved by the institutional review board of the institution. The approved indications for MOWHTO were patients 65 years or younger with isolated medial compartment OA of the knee and varus malalignment. MOWHTO was contraindicated if a patient had symptomatic OA in the lateral compartment or the patellofemoral joint, inflammatory arthritis, flexion contracture of 15° or more, knee range of motion less than 120°, joint instability, or a history of knee joint infection [24]. The patients with isolated medial compartment OA with varus deformity under the age of 65 met the inclusion criteria and were included in the study. The patients with traumatic OA, osteonecrosis, follow-up period less than 1 year, and/or incomplete data were excluded. Thus, four cases from the expert group (two knees with a preoperative surgery history on the affected knee and two knees with follow-up loss) and three cases from the novice group (one knee with a previous operation history, one knee with an infection history, and one knee with follow-up loss) were excluded based on the exclusion criteria. Therefore, 48 knees (*n* = 48 patients) in the expert group and 29 knees (*n* = 29 patients) in the novice group were enrolled for final analysis.

### Surgical technique

Preoperatively, correction angles were determined in all patients via the Dugdale method [23] using weight-bearing full-length hip–ankle radiographs on the Picture Archiving and Communication System (PACS) (PI View STAR; Infinitt, Seoul, Korea) [22]. The correction angle was calculated using the angle formed by two lines in the preoperative plan; the first of these ranged from the center of the hip to the so-called Fujisawa point, including 62.5% of the width of the tibia in the tibia plateau, while the second ranged from the Fujisawa point on the tibial plateau to the center of the ankle joint [25].

**Fig. 1:**
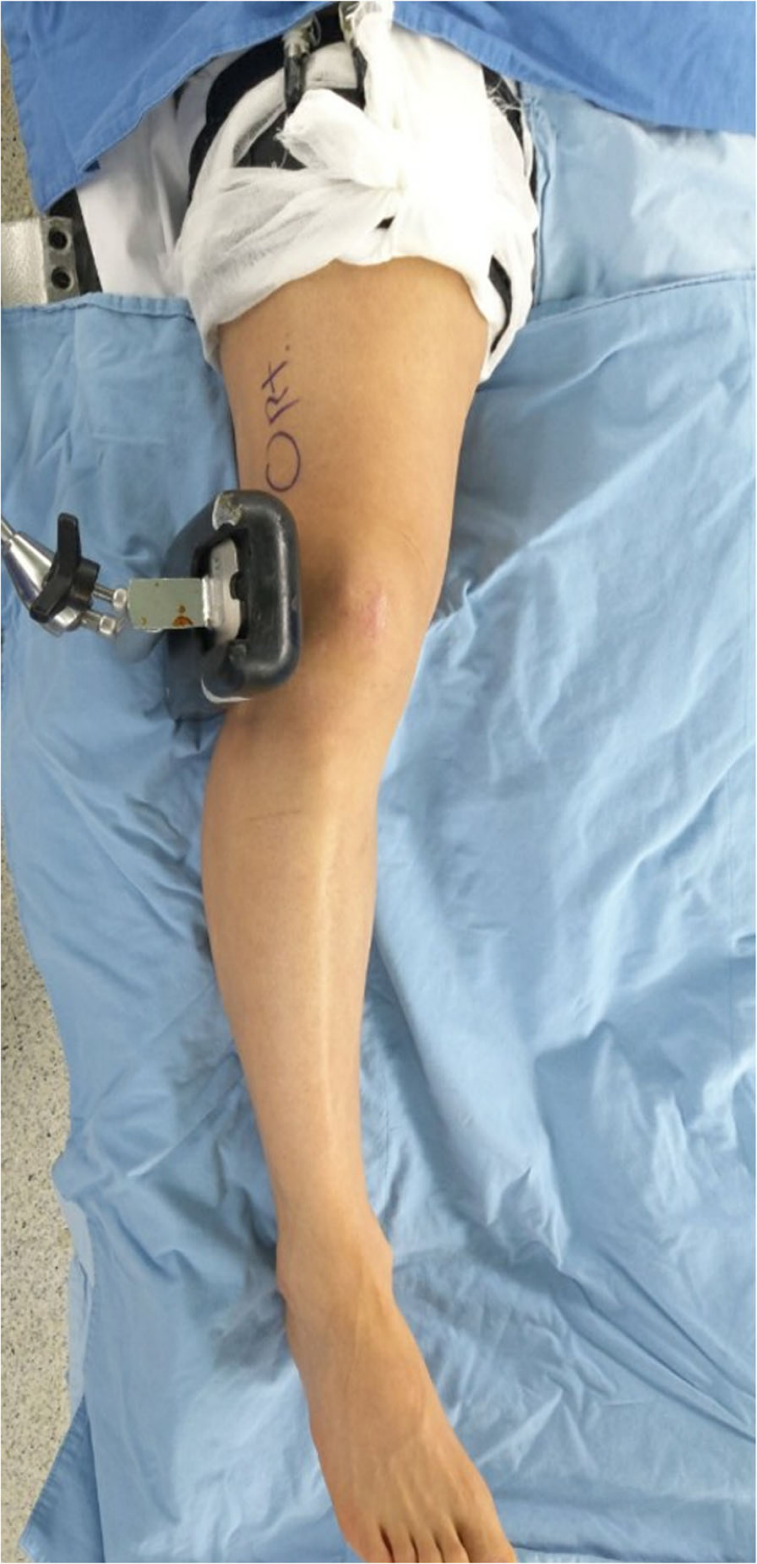
A valgus bar was applied on the table laterally to the operating knee

A valgus bar was applied to the lateral knee joint preoperatively for the “alignment adjustment under valgus stress technique” (Fig. 1). All surgical procedures were performed under general anesthesia with a tourniquet inflated to 300 mmHg. In each case, the pes anserinus was identified and released on the medial side of the proximal tibia. Then, the distal portion of the superficial medial collateral ligament was detached from the tibia using a small elevator. Bi-planar MOWHTO was performed according to a technique developed by the Arbeitsgemeinschaft für Osteosynthesefragen knee expert group [26]. A spreader was inserted into the osteotomy site and the osteotomy site was opened gradually to the planned angle. The opening angle was identified from the angle scale on the spreader [26]. The osteotomy gap was left empty without bone graft.

**Fig. 2:**
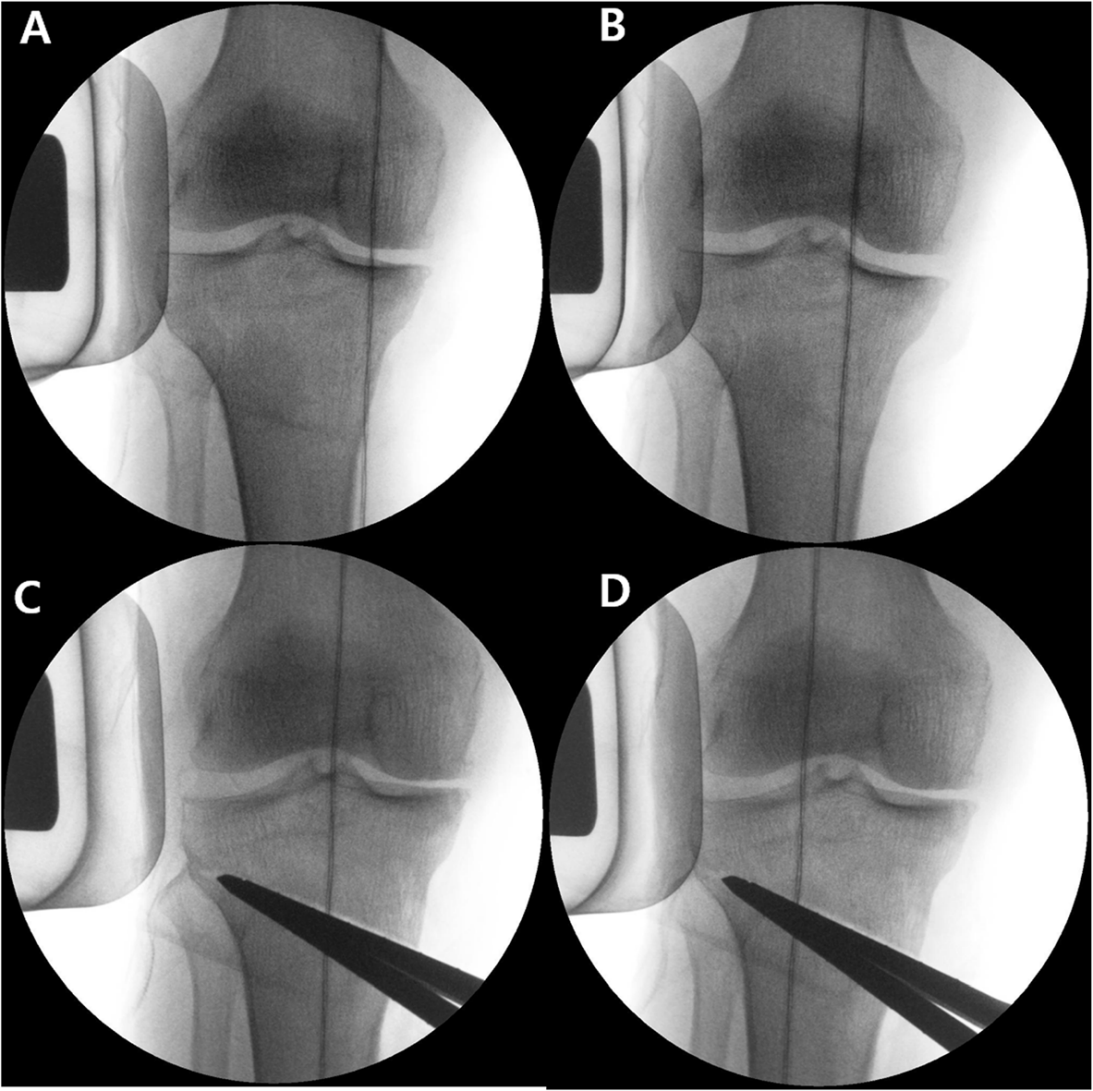
Change in weight bearing line by applying valgus stress, **A** Before applying valgus stress in the preosteotomy state, **B** After applying valgus stress in the preosteotomy state, **C** Before applying valgus stress in the postosteotomy state, **D** After applying valgus stress in the postosteotomy state

**Fig. 3:**
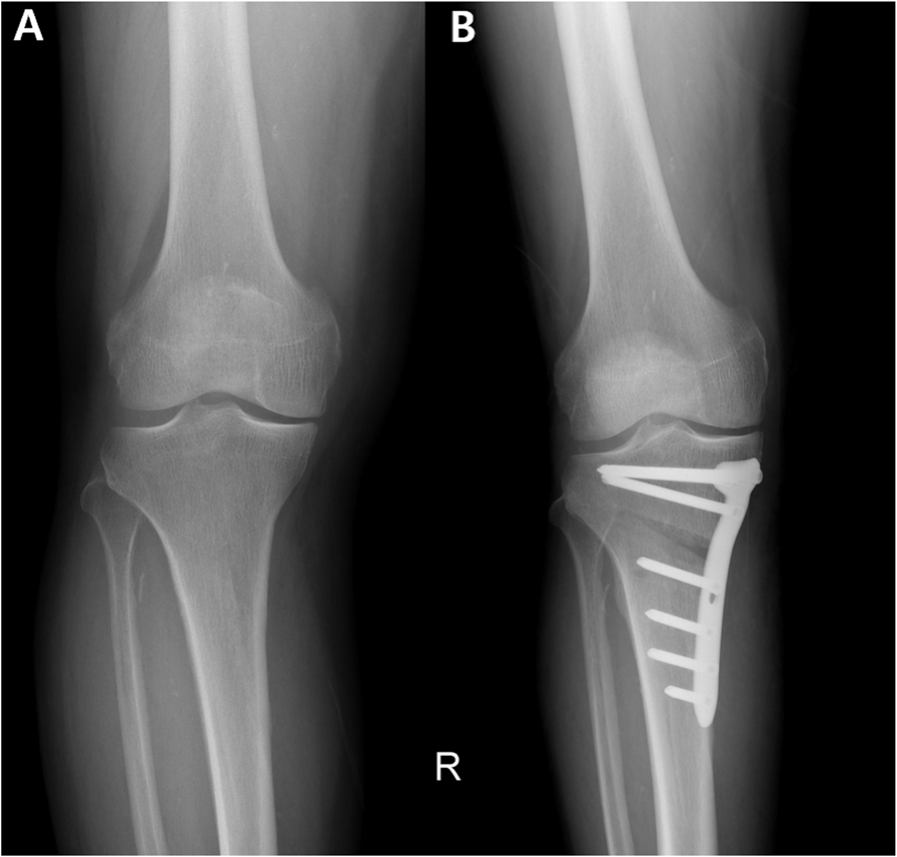
Preoperative (**A**) and postoperative (**B**) radiographs of medial opening wedge high tibial osteotomy

Lower-extremity alignment was assessed by applying valgus stress to the knee joint under an image intensifier following opening of the osteotomy gap as planned by the Dugdale method [22]. Valgus stress was applied to the knee joint with manual maximal force using the valgus bar. By repeating valgus stress several times, the surgeon could feel to what extent valgus stress was appropriate for a specific patient. When valgus stress was applied, the weight-bearing line (WBL) moved to the lateral side (Fig. 2). Then, the alignment was assessed using an electrocautery cable, which passed along the Fujisawa point while applying valgus stress to the knee joint. If the cable passed medial to the Fujisawa point under valgus stress, in a case of undercorrection, the osteotomy gap was opened more to increase the correction angle. On the other hand, in the case of overcorrection, the spreader was closed to reduce the correction angle [22]. Following adjustment under valgus stress, the osteotomy site was fixed with a locking plate (Tomofix; Synthes, Solothurn, Switzerland). (Fig. 3.)

The same postoperative rehabilitation program was applied in both the expert- and novice-performed surgery groups. A quadriceps-setting exercise and continuous passive motion began on the first postoperative day. Partial weight bearing with crutches was allowed from 4 weeks after surgery, and full weight bearing was started 6 weeks after surgery. The protocol was equally applied to all patients including those with underwent meniscal or cartilages procedures.

The radiographic assessment was based on the WBL ratio using weight-bearing full-length hip–ankle radiographs taken preoperatively and at 3 months, 6 months, and 1 year postoperatively. The WBL ratio was defined as the point at which the mechanical axis (MA) passed through the tibial width after drawing a line from the center of the femoral head to the center of the talus dome [15]. The medial and lateral margins of the tibial articular surface were determined as 0 and 100%, respectively. Thus, if the WBL passed through the medial side of the medial edge of the tibia, the value was negative. A PACS was used for radiographic measurements. The acceptable target range of postoperative alignment was a WBL ratio within 62.5% ± 7.5% from the medial edge of the tibia. Meanwhile, an acceptable WBL ratio was defined as a range of 55 to 70%, undercorrection was determined to be a WBL ratio of less than 55%, and overcorrection was defined as that of more than 70% [27]. The accuracy of surgical performance was compared between the expert- and novice-treated groups using the ratio of normocorrection to outliers. Two orthopedic surgeons who were blinded to the intraoperative procedure and the group measured preoperative and postoperative WBL ratios twice at a two-week interval. Each tester was blinded to the other’s measurements and to all patients’ data. The average value of the measurements of the two testers was used. Intraobserver and interobserver reliability were calculated for the reliability of the measurement using the intraclass correlation coefficient (ICC). The intraobserver and interobserver ICC values in this study were greater than 0.8.

**Fig. 4:**
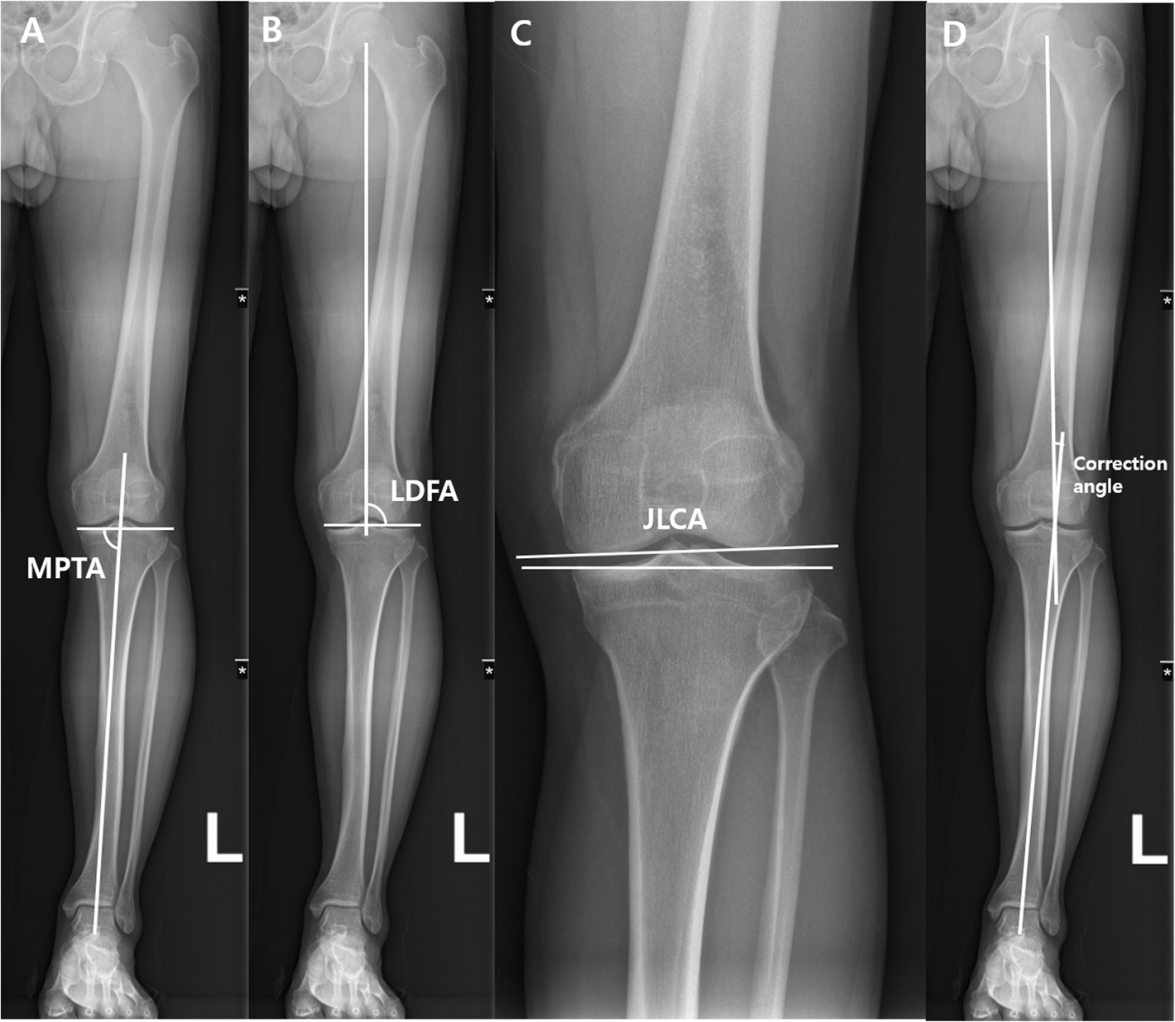
Radiographic parameters for evaluation of coronal alignment and correction angle of the knee using whole leg anteroposterior radiographs. Medial proximal tibial angle (MPTA) (**A**), lateral distal femoral angle (LDFA) (**B**), joint line convergence angle (JLCA) (**C**), correction angle as planned by Dugdale method (**D**)

The MA, medial proximal tibial angle (MPTA), lateral distal femoral angle (LDFA) and JLCA were also measured preoperatively and at 3 months, 6 months, and 1 year postoperatively. The MPTA was defined as the angle that existed between the tibial MA and the articular surface of the proximal tibia. Finally, the LDFA was defined as the angle between the femoral MA and the articular surface of the distal femur [28]. The JLCA was defined as the angle that formed between two articular surfaces of the distal femoral condyle and the tibial plateau [29]. (Fig. 4.)

The knee range of motion (ROM) was measured before and at 1 year after surgery. An orthopedic surgeon who did not participate in the operation measured it using a 60-cm goniometer with the patient in the supine position. Clinical outcomes were evaluated using Western Ontario and McMaster Universities Osteoarthritis Index (WOMAC) scores 1 day before surgery and 1 year after surgery. A WOMAC score with a 15-point improvement based on the minimum clinically important difference (MCID) was assessed at postoperative 1 year [30]. Postoperative complications were also monitored for during the follow-up period.

### Statistical analysis

Demographic data; MA; MPTA; LDFA; JLCA; WBL; and under-, normo-, and overcorrection ratios were compared between the novice and expert groups. The comparison of categorical variables between the two groups was performed using Pearson’s chi-squared test. The analysis of continuous normal distribution data was performed using the Student’s t-test. The Mann–Whitney U test was used for the analysis of the ordinal categorical and non-normal distribution data. The reproducibility of real weight-bearing alignment of the “alignment adjustment under valgus stress technique” was evaluated by comparing the WBLs recorded between during the intraoperative valgus technique and at 1 year postoperatively. ICC values were used for the intra-observer reliability of the radiographic assessments performed by the two blinded orthopedic surgeons. When referring to the results of a previous study [22] and assuming that a 30% significant difference in proportion of outliers excluding normocorrection between the two groups. When alpha was set as 0.05 and beta was 0.80 and the ratio of the two groups was 2:1, 22 patients in the novice group and 45 patients in the expert group were required. Therefore, the present study was considered to be appropriately powered to detect a clinically significant difference with greater than 80% power. The Statistical Package for the Social Sciences version 21.0 (IBM Corporation, Armonk, NY, USA) was used for statistical analyses. A *p*-value of less than 0.05 was considered to be statistically significant.

## Results

There were no significant differences between the two groups in terms of patient characteristics (Table 1). The preoperative MA was varus 6.8° in the expert group and varus 6.4° in the novice group (*p* = 0.536), while the postoperative MA was valgus 2.4° in the expert group and valgus 2.6° in the novice group at 1 year after the operation (*p* > 0.05).

**Table 1:** Comparisons of patient demographics and preoperative deformity data between groups

	Expert group (*n* = 48)	Novice group (*n* = 29)	*p*-value
Demographics			
Age (years)	55.8 ± 7.2	54.8 ± 6.5	0. 528
Sex (% female)	42 (87.5%)	21 (72.4%)	0.129
BMI (kg/m^2^)	25.9 ± 3.8	25.1 ± 3.9	0.344
OA (K-L grade)			0.300
≤ 2	3 (6.3%)	5 (17.2%)	
3	42 (87.5%)	22 (75.9%)	
4	3 (6.3%)	2 (6.9%)	
Additional procedures			
Partial meniscectomy	45 (93.8%)	24 (82.8%)	0.120
Meniscus repair	2 (4.2%)	5 (17.2%)	
Microfractures	44 (91.7%)	26 (89.7%)	0.766
Preoperative mechanical axis (°)	Varus 6.8 ± 23.2	Varus 6.4 ± 2.5	0.536
Preoperative WBL ratio (%)	16.3 ± 12.3	21.3 ± 11.4	0.068
Preoperative JLCA (°)	3.0 ± 1.0	2.6 ± 0.7	0.069
Preoperative MPTA (°)	83.2 ± 2.0	83.3 ± 1.6	0.931
Preoperative LDFA (°)	87.5 ± 2.5	88.3 ± 1.4	0.089
Angle to be corrected by the Dugdale method (°)	10.2 ± 2.7	9.7 ± 2.2	0.337

*BMI* Body mass index, *OA* Osteoarthritis, *K-L* Kellgren–Lawrence, *WBL* Weight-bearing line, *JLCA* Joint line convergence angle, *MPTA* Medial proximal tibial angle, *LDFA* Lateral distal femoral angle

Values are presented as mean ± standard deviation.

The mean preoperative and postoperative one-year WBL ratios were 16.3 and 58.6%, respectively, in the expert group and 21.3 and 60.2%, respectively, in the novice group. No differences in the preoperative and postoperative WBL ratios were observed between the groups (all *p* > 0.05). JLCA also showed no difference between the two groups from before to 1 year after surgery (all *p* > 0.05) (Table 2).

**Table 2:** Comparison of postoperative WBL ratio, HKA angle, and JLCA between the two groups

	Postoperative 3 months			Postoperative 6 months			Postoperative 1 year		
	Expert	Novice	*p*-value	Expert	Novice	*p*-value	Expert	Novice	*p*-value
Postoperative WBL ratio (%)	58.4 ± 7.9	61.5 ± 6.1	0.069	58.0 ± 6.9	60.4 ± 5.3	0.114	57.1 ± 8.4	60.2 ± 5.5	0.089
Postoperative HKA angle	Valgus 2.5 ± 1.7	Valgus 2.7 ± 1.5	0.608	Valgus 2.4 ± 1.7	Valgus 2.7 ± 1.5	0.396	Valgus 2.4 ± 1.9	Valgus 2.6 ± 1.6	0.740
Postoperative JLCA (°)	1.7 ± 0.9	1.4 ± 0.7	0.117	1.8 ± 0.8	1.4 ± 0.8	0.086	1.7 ± 0.9	1.5 ± 1.0	0.453

*WBL* Weight-bearing line, *HKA* Hip–knee–ankle, *JLCA* Joint line convergence angle.

Values are presented as mean ± standard deviation.

**Fig. 5:**
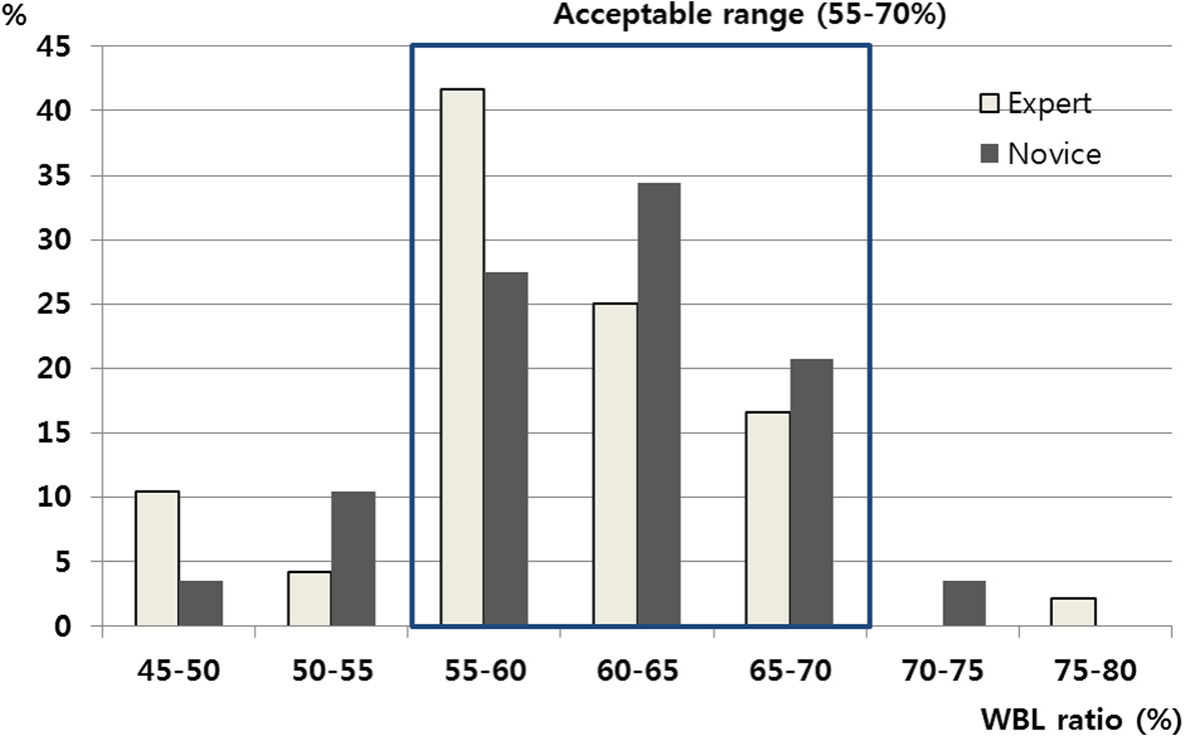
Distribution of the postoperative WBL ratio between the expert and novice groups

The undercorrection rate was 14.6% in the expert group and 13.8% in the novice group at 1 year after surgery, while the overcorrection rate was 2.1% in the expert group and 3.4% in the novice group. The correction accuracy, presented as the ratio of normocorrection to outliers, was 83.3% in the expert group and 82.8% in the novice group. No difference was found during all time points of the follow-up period (all *p* > 0.05) (Table 3 and Fig. 5). The mean WBL ratio before valgus stress was 58.0% (50.0 ~ 68.8%) in the novice group and 55.5% (48.0 ~ 66.0%) in the expert group (*p* > 0.05). After valgus stress, the mean WBL ratio was 63.9% (55.2 ~ 73.2%) in the novice group and 62.3% (55.0 ~ 70.0%) in the expert group (*p* > 0.05). After valgus stress, the WBL was migrated laterally by 5.9% in the novice group and by 6.8% in the expert group, with no difference between the two groups (*p* = 0.199). The reproducibility of the actual weight-bearing alignment of the technique, presented as the WBL difference between the intraoperative period and 1 year after surgery, shifted to the medial side by 2.1% in the expert group and 3.4% in the novice group after surgery (*p* = 0.460). In the novice group, three patients required alignment adjustment after correction. Additional opening of the correction angle was needed in two patients, and further closure of the correction angle was needed in one patient. In the expert group, five patients needed alignment adjustment after correction. An additional increase of correction angle was needed in four patients, and an additional decrease of correction angle was needed in 1 patient.

**Table 3:** Comparison of postoperative WBL ratio

	Expert (*n* = 48)	Novice (*n* = 29)	*p*-value
Postoperative 3 months			0.849
Undercorrection (< 55%)	7 (14.6%)	3 (10.3%)	
Normocorrection (55–70%)	39 (81.3%)	25 (86.2%)	
Overcorrection (> 70%)	2 (4.2%)	1 (3.4%)	
Postoperative 6 months			0.820
Undercorrection (< 55%)	9 (14.6%)	3 (10.3%)	
Normocorrection (55–70%)	38 (83.3%)	25 (86.2%)	
Over-correction (> 70%)	1 (2.1%)	1 (3.4%)	
Postoperative 1 year			0.933
Undercorrection (< 55%)	7 (14.6%)	4 (13.8%)	
Normocorrection (55–70%)	40 (83.3%)	24 (82.8%)	
Overcorrection (> 70%)	1 (2.1%)	1 (3.4%)	
*p*-value	0.971	0.994

The preoperative ROM was 135.4 degrees in the novice group and 134.1 degrees in the expert group. One-year postoperative ROM was 136.5 degrees in the novice group and 135.5 degrees in the expert group, and there was no difference between the two groups (all *p* > 0.05). Preoperatively, there were no differences in the pain, stiffness, or function subscores or the total score of WOMAC (all *p* > 0.05, respectively). At 1 year after surgery, all patients in each group showed significant improvements in WOMAC scores following MOWHTO (all *p* < 0.05), without between-group differences (all *p* > 0.05) (Table 4). The MCID achievement rate was 83.3% in the expert group and 79.2% in the novice group without difference between the two groups (p > 0.05).

**Table 4:** Preoperative and postoperative clinical outcomes

	Preoperative			Postoperative 1 year		
	Expert	Novice	*p*-value	Expert	Novice	*p*-value
WOMAC^a^	53.6 ± 16.1	48.1 ± 12.0	0.165	23.0 ± 12.0	21.6 ± 9.8	0.612
Pain	9.9 ± 3.6	9.7 ± 3.2	0.865	4.0 ± 2.6	3.7 ± 2.5	0.646
Stiffness	4.7 ± 2.1	3.7 ± 2.8	0.079	1.6 ± 1.4	1.5 ± 1.7	0.730
Function	39.0 ± 12.1	34.7 ± 8.9	0.112	17.4 ± 9.1	16.4 ± 6.6	0.628

^a^The WOMAC score

No knee had major complications requiring additional surgery for any reason during the one-year follow-up period. The frequency of lateral hinge fracture was 22.9% (*n* = 11 cases) in the expert group and 20.7% (*n* = 6 cases) in the novice group (*p* = 0.819). Of the 11 cases in the expert group, 10 cases were type 1 fractures and one case was a type 2 fracture [31]. Meanwhile, all six cases in the novice group were type 1 factures. A fixed rehabilitation protocol that allowed weight bearing at 4 weeks after surgery was applied to patients with lateral hinge fractures. There was no case of correction loss, delayed union, or nonunion in either group.

## Discussion

The most important finding of this study was that a novice surgeon could achieve similar acceptable surgical accuracy to that of an expert surgeon in finding the acceptable correction range by applying “alignment adjustment under valgus stress technique” during MOWHTO.

For a novice surgeon, reducing the learning curve is very important for providing adequate health care to patients [12, 13]. Since the frequency of MOWHTO is less than that of total knee arthroplasty or anterior cruciate ligament reconstruction, on an occasional basis, more time spent performing MOWHTO procedures may be required to achieve adequate surgical competency [32–34]. In MOWHTO, the paramount factors for satisfactory postoperative clinical outcomes are appropriate patient selection [35] and execution of correction [8]. Lee et al. investigated the learning curve of MOWHTO using 100 consecutive cases [13]; to prevent undercorrection, 27 MOWHTO cases were required, while, in the case of overcorrection, it was difficult to prevent such adequately even by 100 cases [13]. It has been reported that joint laxity, especially on the lateral side, could affect lower-extremity alignment following HTO [2, 14]. The surgical pearls for successful MOWHTO include representation of weight-bearing alignment of the patient, who is in the supine position during surgery [2, 14, 36, 37]. In fact, there have been many studies examining the relationship between factors related to joint laxity, including JLCA, to predict the actual standing alignment of MOWHTO patients [2, 14, 36, 37]. However, there continue to be limitations to the prediction of postoperative alignment through radiation measurements related to joint laxity before surgery [2, 14, 36, 37]. Therefore, it is necessary to reproduce the alignment of the standing position while reflecting the joint laxity that differs for each individual in the actual surgical field. In the “alignment adjustment under valgus stress technique” protocol used in this study, the WBL was shifted to the lateral side by applying valgus stress to offset the joint laxity and the amount of adjustment varied from patient to patient. The soft tissue imbalance through valgus-varus stress was imaged before surgery. The degree of soft tissue imbalance through preoperative stress images could be useful to predict joint laxity before surgery [29, 38–40]. The advantage of applying valgus stress intraoperatively is to estimate the weight-bearing alignment after correction. By experiencing this technique, we found that the osteotomy site did not open when subjected to valgus stress and only the alignment moved to the lateral side. By using this technique, the novice surgeon could achieve a similar degree of accuracy in surgical performance as that of an expert surgeon. In addition, the degree of reproducibility of the actual standing alignment of the “alignment adjustment under valgus stress technique” also showed no difference between the two groups.

It is difficult to achieve postoperative alignment within the acceptable range following MOWHTO [13, 41–43]. Miniaci et al. [42] stated that when a Fujisawa point of ±10% of WBL ratio after HTO is set as an acceptable range, only 50% falls into this range. Elsewhere, Marti et al. [41] reported that the accuracy of MOWHTO was also about 50%, with 31% of cases showing undercorrection and 19% showing overcorrection. El-Azab et al. [44] reported an undercorrection rate of 8% and an overcorrection rate of 6% when a WBL range of 50 to 70% was set as an acceptable range. In the study of Lee et al. [13], when the acceptable range for WBL was set as 57 to 67%, a total of 61% of outliers were reported, stratified as 23% of cases with undercorrection and 38% of cases with overcorrection. When Stanley et al. [43] set 48 to 68% as an acceptable range, the conventional technique showed 22% of cases had undercorrection and 15% had overcorrection. Kim et al. [22] reported that the outlier was about 50% before using the intraoperative valgus stress technique, yet decreased significantly by about 10% after the intraoperative valgus technique was adopted. In this study, rates of undercorrection and overcorrection were 13.8 and 3.4% in the novice group and 14.6 and 2.1% in the expert group. Both expert and novice surgeons had acceptable levels of accuracy relative to in previous studies [13, 22, 41–43]. In particular, according to the study by Lee et al. [13], it was difficult to prevent overcorrection even if surgical competency with HTO was accumulated. However, in our study, it could be of great significance that the frequency of overcorrection was very low for both novice and expert surgeons when using the intraoperative valgus technique.

Various methods have been reported to obtain an appropriate alignment following HTO, such as full-length radiographs of the lower extremity, imaging software, electrocautery cables, grids, fluoroscopy, and a navigation system [15–17, 19, 21, 45]. However, since the confirmation of alignment during surgery is performed in the supine position, it cannot be recognized without fault as the alignment of the postoperative weight-bearing position. Sim et al. [46], as a result of examining patients who underwent MOWHTO, found that there were differences in the MA and WBL on standing and supine radiographs. Specifically, when performing weight-bearing assessments, the WBL ratio moved to 12% lateral and the FTA angle increased by 1.7 degrees in valgus. Therefore, the authors applied an axial compression force to the heel to reproduce weight-bearing during surgery [46]. Marti et al. [41] also used a push orthoradiography technique with an axial compression force applied to reproduce the weight-bearing status. Kim et al. [22] reported that the weight-bearing status was reproduced through the intraoperative valgus stress technique and that the WBL ratio of 9.6% moved laterally through the valgus force. In this study, after applying valgus stress, WBL moved laterally by 5.9% in the novice group and 6.8% in the expert group, which means that the technique shifted the baseline WBL to a more lateral point, which might have been especially effective in reducing overcorrection outliers. The novice surgeon had outliers (under- and overcorrection) of 17.2%, which were similar to that of 16.7% among expert surgeons, and showed similar outliers to that of 12% reported by Kim et al. [22].

The current study had several limitations. First, the proportion of women in both groups was more than 90%. It is a well-known fact that the sex ratio of women in knee osteoarthritis is high in Korea [40, 47, 48]. Second, application of valgus stress using a valgus bar could not reproduce exactly the weight-bearing status, and the degree of valgus stress force could be subjective. However, by measuring valgus stress several times, the surgeon could posit to what extent valgus stress was appropriate for a specific patient. Although both expert and novice surgeons obtained normo-corrections in greater than 80% of patients with this simple technique, the method is subjective. Third, the follow-up period was as short as 1 year. Comparisons between the two groups during longer-term follow-up can provide more accurate results. Fourth, various confounding factors, such as spatial ability, innate skill, and previous experience, can influence the learning curve in the surgery, but all of these factors were not considered. Therefore, there are limitations in generalizing the results of one novice surgeon to other surgeons. Fifth, although there were no significant differences in the rates of additional procedures such as meniscal or cartilage surgery between the two groups, additional procedures during MOWHTO could affect the clinical outcomes [7, 47, 49, 50]. Finally, a number of variables were measured on radiographs. Therefore, there may be differences attributed to variable radiographic techniques or measurement methods. In this study, to reduce such errors, the full-length radiograph was measured with the patella and both feet in a forward posture and the full extension of the knee joint was applied to reduce the error as much as possible.

Despite these shortcomings, our study revealed that even a novice surgeon in the learning period could obtain an appropriate level of alignment accuracy when implementing MOWHTO through a method that can be easily applied in the operating room. For clinical relevance, as shown by the results of the current study, the use of the “alignment adjustment under valgus stress technique” in the practice of MOWHTO during the learning period of a novice surgeon was reliable and reproducible enough to reduce the learning curve required to perform accurate correction.

## Conclusion

A novice surgeon could have a similar level of surgical accuracy in alignment correction to that of an expert by using the “alignment adjustment under valgus stress technique” protocol during MOWHTO.

## Data Availability

The datasets used and analyzed during the current study available are from the corresponding author upon reasonable request.

## Abbreviations

MOWHTO: Medial opening-wedge high tibial osteotomy
WOMAC: Western Ontario and McMaster Universities Osteoarthritis Index
OA: Osteoarthritis
